# 

**Published:** 1904-04

**Authors:** 


### GEORGIA SOCIOLOGICAL SOCIETY.

THE third annual meeting of the Georgia Sociological Society will be held in Atlanta, May 10, 11, 12. While the program has not yet been completed, the committee is able to announce that the prospects for an interesting series of papers are altogether favorable. Among the speakers who have given their acceptance are Drs. Kime, Hiers, Powell, Olmsted, Pinckney, Stirling. Other medical men, representing the different sections of the State, will be invited to present papers. Rev. Len. G. Broughton will read a paper on "Juvenile Courts," and Judge Nash Broyles will speak of his experiences as recorder of Atlanta. Mr. J. C. Logan will discuss the "Need of a Free Legal Aid Society." Mr. Harry Schlesinger will discuss the "Relation of the Employer and Employee." The work of this society appeals to progressive men and women in all the walks of life. The organization is simple and the membership fee is one dollar per annum. Those desiring membership will communicate with the secretary, Rev. C. A. Langston, 530 Spring Street, Atlanta, Ga.

**This Journal and Atlanta Semi-weekly Journal one year for $1.25 cash in advance. No checks. Strictly to new subscribers.**

***NOTICE.— This Journal and the International Journal of Surgery one year for $1.00 cash, to new subscribers. No out-of-town checks accepted unless 10c. is added for cost of cashing. Strictly to new subscribers.***

**This Journal and Atlanta Semi-weekly Journal one year for $1.25, cash in advance. No checks. Strictly to new subscribers.**

